# The impact of root end filling material type and the application of bone graft on healing of periapical tissues after endodontic microsurgery (a clinical randomized controlled trial)

**DOI:** 10.1038/s41598-024-66033-w

**Published:** 2024-10-25

**Authors:** Hesham Mohamed Salah, Ahmed Abdel Rahman Hashem, Tarek Mustafa, Amgad Hassan Soliman, Mustafa Khallaf, Haseeb Haddadeen

**Affiliations:** 1 Endodontic Department, Faculty of Dentistry, Future University in Egypt, New Cairo City, Egypt; 2 Ain Shams University, Cairo, Egypt; 3 Cleveland Dental Institute, Cleveland, OH USA; 4 Endodontic Department, Faculty of Dentistry, Ain Shams University, Cairo, Egypt; 5 Faculty of Dentistry, Cairo – Boys, Al Azhar University, Cairo, Egypt; 6 College of Arts and Sciences & College of Medicine, The Ohio State University, Columbus, USA

**Keywords:** Apicoectomy, Bone graft, Endodontic surgery, MTA, TotalFill, Apical lesions, Outcomes research, Dental diseases

## Abstract

To evaluate the effect of combining different bioactive root-end filling materials with composite bone graft (xenogeneic mixed with autogenous bone fragments) on the healing process of periapical tissues after endodontic micro-surgery procedure. In this triple-blinded clinical trial, 56 patients were divided into 2 main groups (28 each) according to the root-end filling material and 2 subgroups according to the presence or absence of the composite bone graft material. Group I: MTA root-end filling (n = 28) in which there are Sub-group A: without bone graft (n = 14) and Sub-group B: with composite bone graft (n = 14). Group II: TotalFill root-end filling (n = 28) in which there are Sub-group A: without bone graft (n = 14) and Sub-group B: with composite bone graft (n = 14). Healthy patients whose ages range from 20 to 50 years with small-to-medium size radiolucency in CBCT related to single-rooted maxillary teeth were included in this study. Patients were assigned a number starting from 1 to 56 and were randomly allocated to four test groups (2 main groups and 2 sub-groups) following simple randomization procedure guidelines described by IBM SPSS V23 (IBM USA) statistical analysis software. This trial was triple-blind where the patient, the outcome assessors, and the main operator were blinded to the applied intervention. Every patient was evaluated clinically and by CBCT at two main observation periods: presurgical and 12-month post-operative. They were also examined and evaluated clinically and radiographically through periapical x-rays after one week, three, and six months. Statistical analysis was performed with IBM SPSS Statistics for Windows Version 23.0. Armonk, NY: IBM Corp. Of the 56 patients enrolled in the study, 49 patients were available for the final analysis. All groups showed no statistically significant differences with regard to healing or success rates at the 12-month follow-up mark. No adverse effects were encountered. Results showed that high success rates were achieved using MTA and TotalFill in the healing of periapical lesions after endodontic surgery. The addition of bone graft in small-to-medium size lesions did not affect the success rate of endodontic surgeries.

## Introduction

Post-treatment disease is a counterproductive outcome following endodontic treatment. It could occur due to iatrogenic errors or mishaps during treatment^1^. Examples can be perforations, ledges, separated instruments, coronal leakage, and/or incomplete cleaning of root canal space. Moreover, over-instrumentation of the canal may cause leaky apical seal^2^. It can also happen because of persistent periapical infection, true cystic lesions (independent from the canal system), foreign body reactions, and cracks or fractures to the root structure^3^. When faced with post-treatment disease, the clinician is forced to resort to retreatment options. These options would include non-surgical endodontic retreatment, endodontic surgical intervention, or extraction and replacement with implants or prosthetic options^4,5^.

Endodontic surgical procedures can be a treatment for teeth diagnosed with persistent periapical pathosis, when nonsurgical retreatment is impractical or unlikely to improve results of previous treatment^6,7^. Furthermore, it is also indicated when a biopsy is needed^3^. The radicular resection routinely accompanying this procedure results in exposure of root dentin and root canal filling material^8^. The placement of a root-end filling, following radicular apical resection, is important to seal the root canal system and support bone regeneration^9^. Bioactive root-end filling materials, such as mineral trioxide aggregate (MTA), showed superior healing of the periapical defect compared to teeth treated solely by smoothing of the gutta-percha root filling^10,11^.

MTA is considered the first calcium silicate-based cement (CSC) to appear in endodontic practice procedures that involve hard tissue repair^11^. Despite the superiority of MTA, when compared to its predeceasing endodontic repair cements, it had certain drawbacks related to its clinical use. Such drawbacks include poor handling properties and long setting time, especially for early generations. In addition, the presence of trace toxic elements in the composition, difficulty in retrieval, tendency for tooth discoloration, and high cost are other types of shortcomings^10,11^.

CSCs are constantly being improved and developed to overcome the limitations of MTA^10–12^. While recent products show promise, using alternatives to the gold standard requires further research to support these bold assumptions^13^. FKG introduced a new CSC material: TotalFill. The material is premixed to avoid human mixing errors. It is produced in many consistencies to suit the professional and medicinal needs of clinicians^14^. The company claims its superiority to older generations of CSCs in terms of biocompatibility and bioactivity. They also claim that it has an almost diminished capacity to cause discoloration. Nonetheless, the product has superior handling properties and the setting time claimed by the company is only 2 h^15^.

Successful healing after endodontic surgery depends not only on the bacteria-tight seal of the root canal system with root-end filling but also on the deficiencies of the periapical and marginal bone tissue adjacent to the lesion^16^. The bone replacement materials should be biocompatible and should support the rate of osteogenesis^16^. Full bony regeneration to maximum horizontal potential and subsequent long-term retention has long been a desired result sought by the dental practitioner^17^.

Guided tissue regeneration (GTR) techniques have been suggested as an adjunct to endodontic surgical procedures with the goal of improving the quality of bone healing^18^. Studies evaluating the merits of GTR on the outcome of endodontic surgery are generally diverse with regards to treatment protocols, follow-up periods, and exclusion criteria deriving conflicting results^19^.

Both the isolated use of bone replacement analogue and the combined use of membranes alongside bone replacement analogue could improve the outcome of the apical surgery^16,18–21^. Nevertheless, the use of barrier membranes alone failed to show an impact on the rate of healing in 4-walled defects. Additionally, the use of GTR in large and through-and-through lesions is favorable^22^.

Our study aimed to evaluate the effect of combining different bioactive root-end filling materials with composite bone graft on the healing of periapical tissues after endodontic surgeries. In this study, the null hypothesis was the absence of statistically significant differences between the gold standard MTA and the newer CSC-based pre-mixed TotalFill putty. It was also believed that the addition of composite bone graft in small-to-medium periapical lesions would not affect the outcome of the procedure.

## Materials and methods

### Ethical considerations

This study was approved by the ethics committee of the Faculty of Dentistry, Ain Shams University, Cairo, Egypt (FDASU-RecID041910). The study protocol was first registered on (13/07/2023) at clinical trials website (http://www.clinicaltrials.gov Identifier: NCT05943769). The study was carried out in accordance with the World Medical Association Declaration of Helsinki (2008).

### Trial design

**Figure 1:**
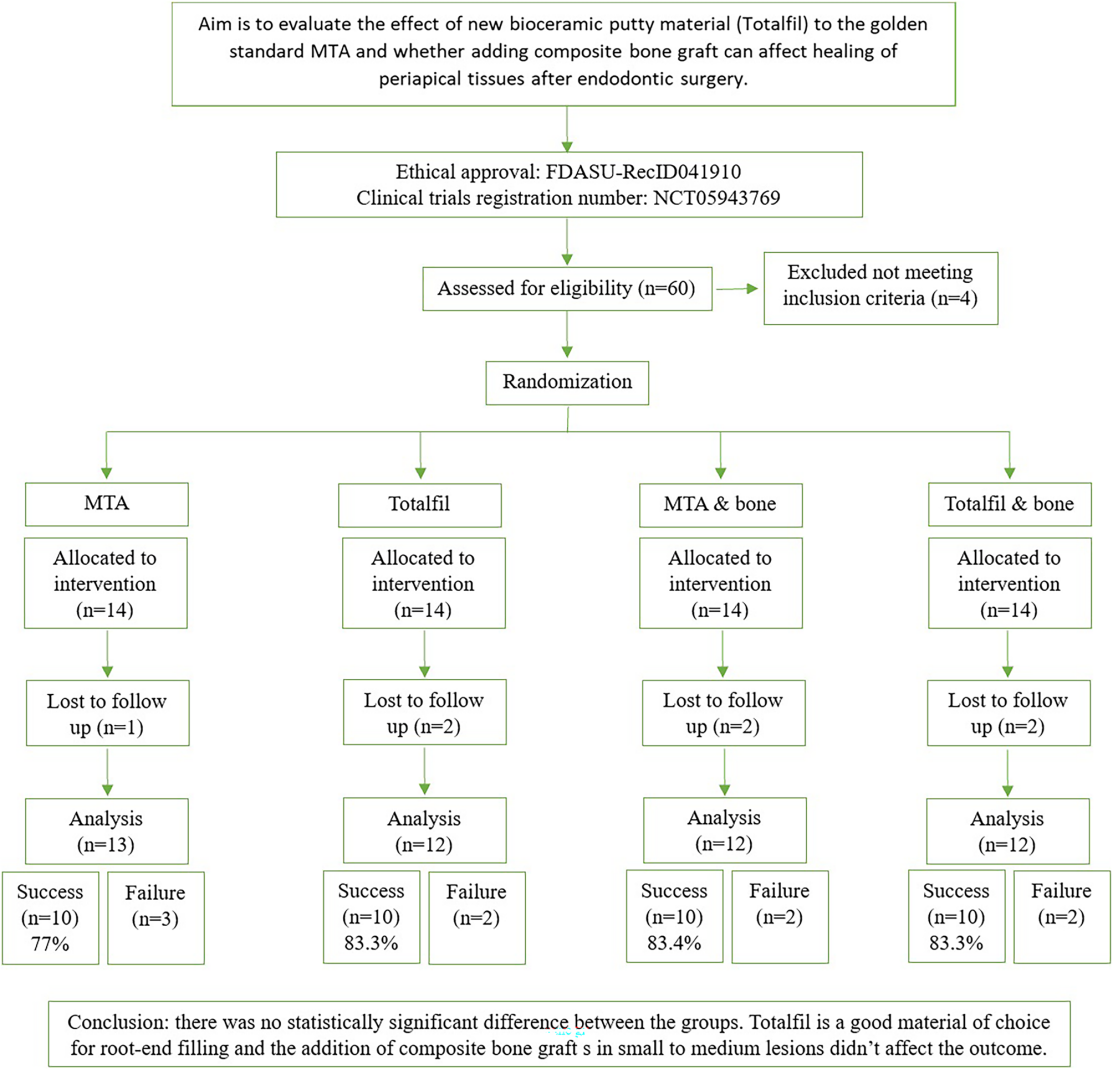
Study Flowchart.

This randomized clinical trial (RCT) conformed to the consolidation standards of The Preferred Reporting Items for Randomized Trials in Endodontics 2020 guidelines (PRIRATE), which proposes a checklist developed using the Consolidated Standards of Reporting Trials and Clinical and Laboratory Images in Publication guidelines. Figure 1 illustrates the study design.

### Sample size calculation

The sample size calculation was based on the results of Tobon et al.^23^ and the assumption that a 50% reduction in lesion size is clinically significant since no relevant literature was found regarding the use of grafts. This sample size calculation is for a 2 × 2 fixed effects analysis of variance. The first factor (Material) included 2 levels, and the second factor (Use of graft) included 2 levels. Using alpha (α) level of (5%) and Beta (β) level of (20%) i.e., power = 80%; the minimum estimated sample size was 12 cases per cell for a total of 48 cases. To compensate for the drop-out rate of 20%, the sample size was increased to 56 cases (14 cases per cell). Sample size calculation was performed using IBM^®^ SPSS^®^ Sample Power^®^ Release 3.0.1.

### Sample selection

After gaining the approval of the local ethical committee (FDASU-RecID041910), 56 patients from the outpatient endodontics clinic located at the Faculty of Dentistry, Ain Shams University were diagnosed by clinical and radiographic means to ensure they fulfilled the set criteria of inclusion.

Inclusion criteria:

- Participating patients were free from any diseases affecting bone healing.
- Ages ranged between 20 and 45 years.
- All patients had generally good oral hygiene.
- Lesions were related to single-rooted endodontically treated maxillary teeth, where different orthograde root canal treatment options failed.
- Lesion sizes ranged from small-to-moderate size (up to 3 cm in highest diameter at any axis in CBCT).

Exclusion criteria:

- Teeth showing apico-marginal defects or teeth suffering from concomitant or true combined periodontal and endodontic pathologies (periodontal pockets and/or mobility).
- Cases with previous endodontic surgery (otherwise known as REsurgery cases) which include apicectomies, root resections, and amputations.
- Cases showing root fractures.

Periapical radiographs and CBCTs were taken to estimate the size of the apical lesion and the condition of the affected teeth. CBCT images were acquired using Acteon X MIND TRIUM CBCT UNIT. The CBCT unit was set at 60-90 kVp, 1-14 mA, with an examination time of 38 seconds. A fixed field of view of 80 x 80 mm was chosen, and the voxel size was set to 100 µm.

### Classification of patients

The patients (n = 56) were divided into 4 groups (n = 14) according to the root-end filling material used with or without the addition of composite bone graft.

Group I: MTA root-end filling (n = 14).

Group II: TotalFill root-end filling (n = 14).

Group III: MTA root-end filling with the addition of composite bone graft (n = 14).

Group IV: TotalFill root-end filling with the addition of composite bone graft (n = 14).

### Randomization

The patients were assigned to a number starting from 1 to 56 and were randomly allocated to one of four test groups (2 main groups and 2 sub-groups) following a simple randomization procedure, using IBM SPSS V23 (IBM, USA) statistical analysis software. Informed consent was obtained from all subjects or their legal guardian(s) for participation in the study.

### Root canal treatment to adjacent teeth involved in the lesion

The tooth to be treated was anesthetized, followed by rubber dam isolation, then access cavity preparation. As for the pre-surgical phase of the study, the root canal treatment procedure was done in a crown-down technique using Protaper Universal rotary system. The crown-down technique has the advantages of early organic debris removal, a straighter access to the apex of canals with a degree of curvature, the creation of a reservoir for irrigating solutions, and enhanced precision in maintaining accurate working length and apical size^24^. In cases of root canal treated teeth, retreatment was carried out, which is suggested to increase success rate by reducing the bacterial load and improving the seal^25^.

During cleaning and shaping, irrigation was performed using 2.5% NaOCl. At this concentration, NaOCl was able to be effective as an antibacterial agent and perform its organic tissue-solving capacity, while limiting its cytotoxic effect due to the direct relation between cytotoxicity and concentration. The application time was 1-min, as no significant statistical difference was found between 1-, 3-, and 5-min application of NaOCl^26^. Smear layer was removed using 3 ml of 2.5% NaOCl for 3 min followed by 3 ml of 17% EDTA solution for 1 minute^27,28^.

Warm vertical compaction technique (Fast-Pact and Fast-Fill, Eighteeth, China) with gutta-percha in conjunction with resin-based root canal sealer (ADSEAL, Meta Biomed, Republic of Korea) was used for obturation of the canals, as it showed a better sealing of canal spaces and slightly better outcomes than the conventional lateral compaction technique^29^. Postoperative radiographs were taken to confirm the density and length of the fill.

### Periapical surgery procedure

As for the surgical part, premedicating the patients with anti-inflammatories (Ibuprofen 600 mg orally 1 h preoperative) was a trial to minimize postoperative inflammation and pain^30^. This study was applied on anterior single-rooted teeth due to their high incidence of trauma with sequelae of pulp necrosis and apical periodontitis. This decision was made to limit variables by avoiding the complicated root canal systems of posterior teeth, which could affect the results due to multiple branching between the canals.

**Figure 2:**
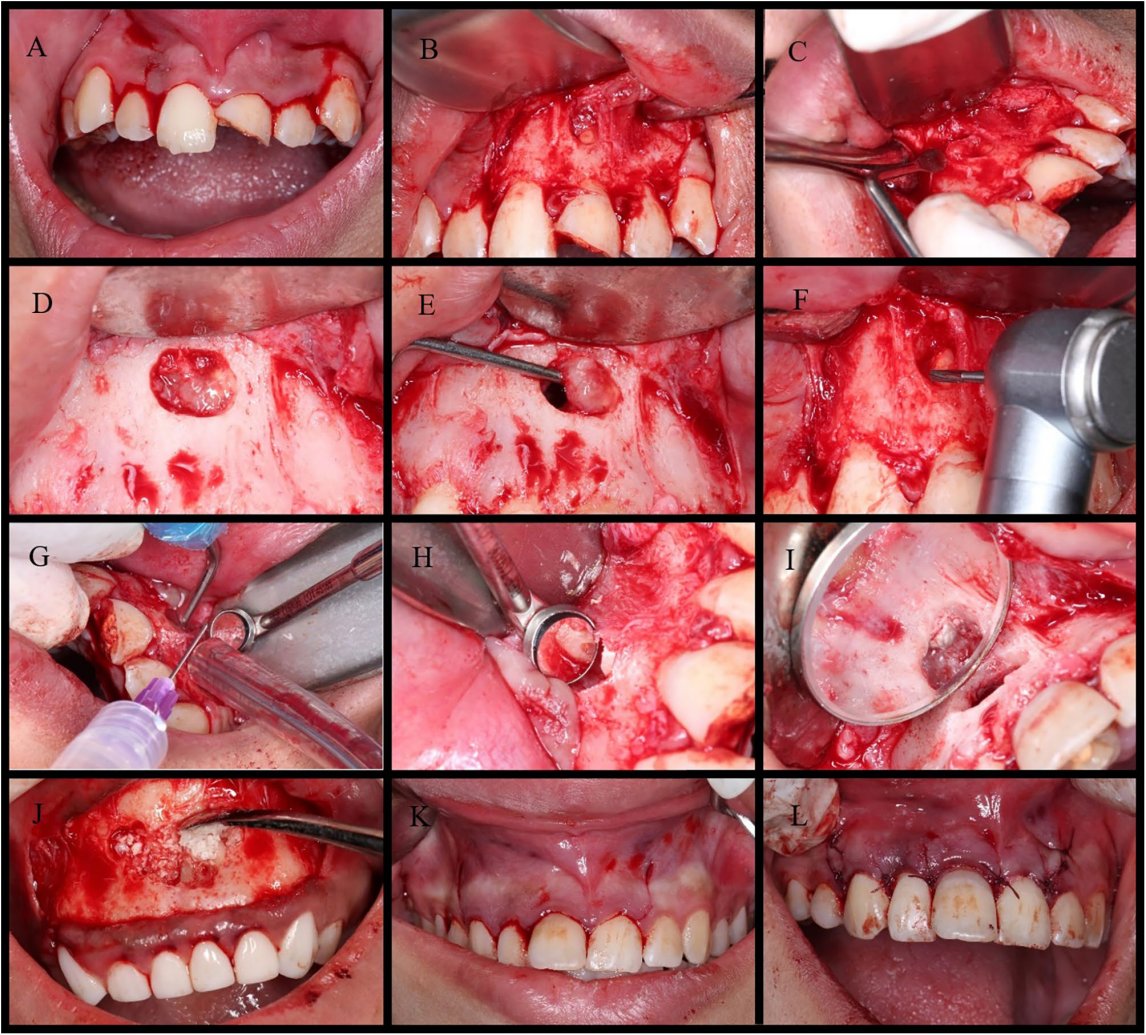
Periapical surgery procedure; (**A**) Incision, (**B**) Flap elevation, (**C**) Osteotomy procedure, (**D**) Complete osteotomy, (**E**) Enucleation, (**F**) Root-end resection. (**G**) Root-end preparation, (**H**) Root-end preparation completed, (**I**) Root-end filling, (**J**) Bone graft placement, (**K**) Flap approximation, (**L**) Sutured flap.

A three-incision-line, full-thickness, mucoperiosteal flap was performed and reflected, providing easy access to the pathological defect and surgical field. This flap design minimizes stress on tissues and allows for ease when reapproximating the flap to its original position. Retraction was done using a Minnesota retractor (Hu-Friedy) that rested on sound cortical bone. This is illustrated in Fig. 2A and B.

The magnification provided by the DOM allowed for a very conservative osteotomy, performed under sterile saline cooling with a plastic syringe (Fig. 2C and D). Several studies reported a correlation between a smaller osteotomy and faster healing^31^.

In cases where the cortical plate is perforated, the osteotomy site is identified with a micro-explorer under the microscope. In cases with sound cortical bone, the tooth length was measured using CBCT software, providing a precise location of the radicular apex position and facilitating the osteotomy site planning.

All granulomatous tissue surrounding the apex was removed to gain access and minimize hemorrhage. Enucleation was done using the concave side of a bone curette (Hu-Friedy) (Fig. 2E).

Root-end resection was performed by a size-three, long shank, tapered round-end abrasive, mounted on an Impact Air 45 handpiece (Vista Apex, Wisconsin, USA), eliminating the possibility of air emphysema. Under copious saline irrigation, 3 mm from the root apex was resected with a 0 to 10-degree bevel (Fig. 2F). Constant cooling minimized heat generation and crack formation, which can occur in dentine during cutting. The cutting was performed perpendicular to the long axis of the root, without any bevel, allowing better conservation of root structure and a higher postoperative crown-to-root ratio while removing most apical ramifications.

Long bevels require the removal of excessive root structure, which causes significant damage to the tissues that the surgery aims to save, such as buccal bone and root. Diagonal resection results in buccal bone removal along with a large area of the root, causing a large osteotomy. Another consideration for the 0° to 10° bevel is that the cavo-surface marginal dimensions of the preparation would be considerably decreased, allowing a better marginal seal^32^. Kim and Kratchman^5^ suggested that at least 3 mm of the root-end must be removed during radicular resection because it contains 98% of the apical ramifications and 93% of the lateral canals.

Retro-cavities were prepared using a specially designed ultrasonic retro-tip AS3D, mounted on a piezoelectric ultrasonic device (Satelec, Acteon), with copious cooling to avoid overheating (Fig. 2G and H). Preparation was done 3 mm in depth, parallel to the long axis of the root, as their walls coincided with the anatomical outline of the pulp space. The use of contra-angled retro-tips facilitated root-end preparation, aiding in access and providing superior control. The ultrasonic unit was adjusted to low intensity to minimize the possibility of dentinal cracks, as stated by Calzonetti et al.^33^. Ultrasonic tips were used in a light, sweeping motion with short forward/backward and upward/downward strokes, resulting in effective cutting action. Interrupted strokes showed more effective cutting than continuous pressure on the dentin surface.

All procedures up to the apicectomy point were performed by the same operator. For blinding purposes, the retro-filling material type was placed, with or without composite bone graft, together with flap repositioning and suturing, according to patient allocation by another operator. Root-end filling material was placed into the retro-cavities according to the allocated group, in one to three increments, and condensed by a suitably sized endodontic retro-plugger instrument (Fig. 2I).

If the patient was allocated to a group requiring a bone graft, a bone scraper was used to obtain autogenous bone particles from healthy bone surrounding the osteotomy. It was then mixed with xenogeneic bone particles (Bio-Oss, Geistlich, Geistlich Pharma, UK) at a ratio 0.5:1 to combine the best properties of both graft types^34^. Before closure, this composite graft was loaded in the osteotomy (Fig. 2J). The mucoperiosteal flaps were re-approximated, compressed, and stabilized before suturing (Fig. 2K). The sutures used were 4.0 polypropylene non-resorbable sutures to achieve primary closure of the wound, as they have low tissue reaction (Fig. 2L). A gentle pressure pack was postoperatively placed over the flap site for several minutes to minimize hematoma formation and enhance the reattachment of soft tissues to the underlying bone, ensuring homeostasis.

### Evaluation

**Figure 3:**
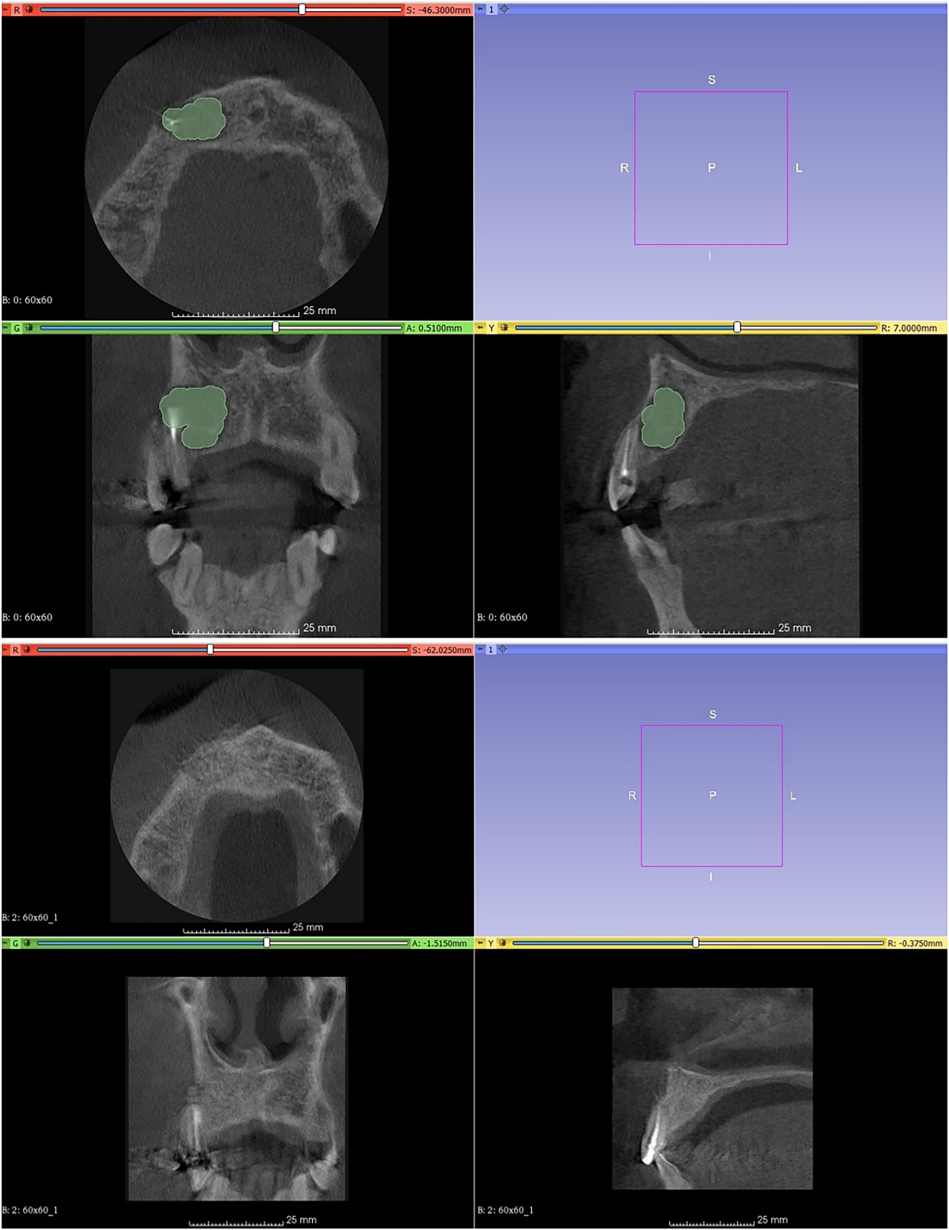
Measurements of volumetric changes preoperative and on follow up.

**Figure 4:**
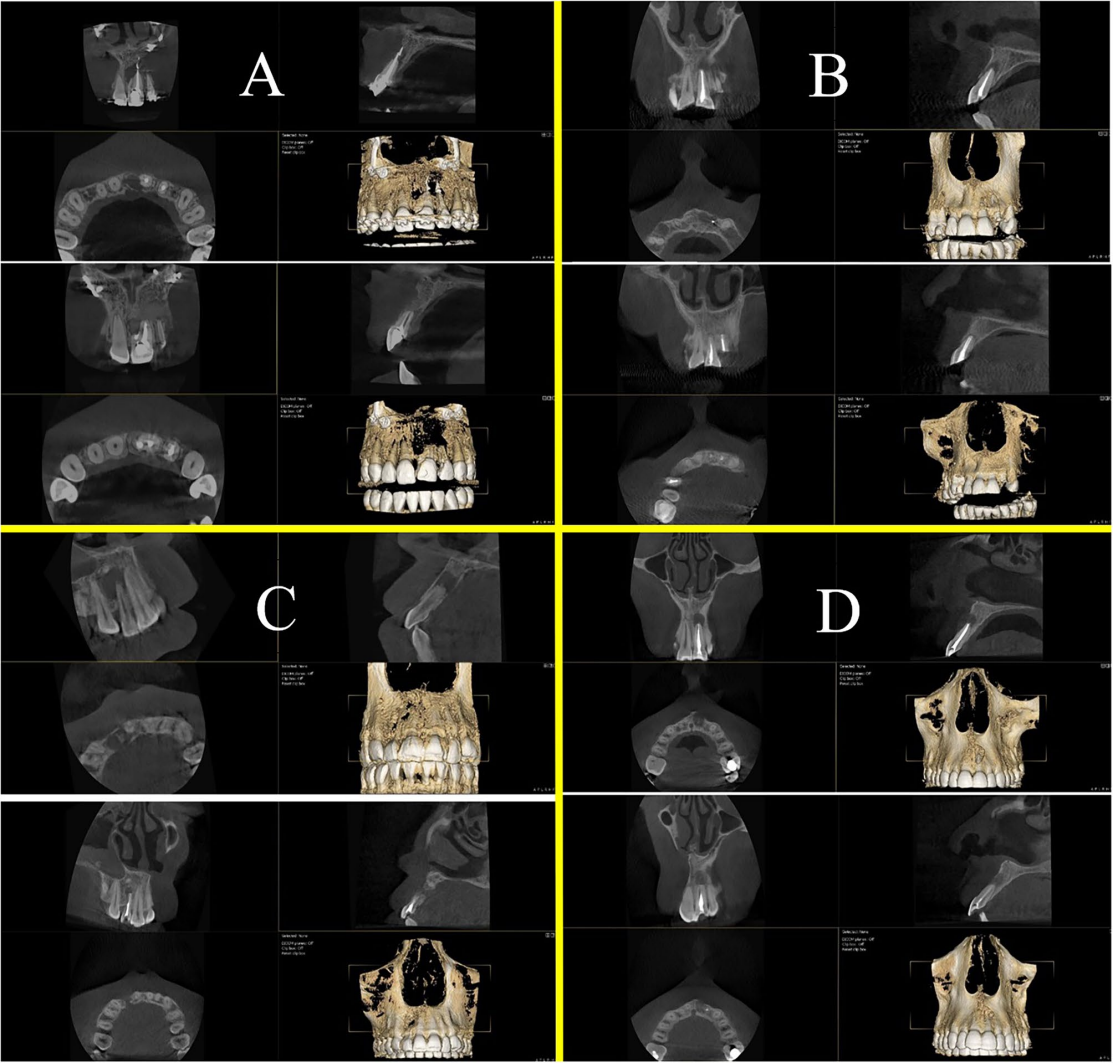
Examples of (**A**) Complete healing, (**B**) Limited healing, (**C**) Uncertain healing, and (**D**) Unsatisfactory healing.

Every patient underwent a clinical examination performed by an experienced, independent, blinded clinician (TM), who did not have any information about the treatment group. Clinical criteria for healing mainly constituted the absence of any signs and symptoms of periapical diseases. At the end of each observation period, all patients were examined for pain, edema, or infection. Radiographic examinations were similarly conducted by an experienced, independent, blinded investigator (AH) with no clinical data available to the radiographic examiner. Periapical bone healing was evaluated by comparing pre-operative and 12-month postoperative CBCTs, measuring the volumetric changes in the bone lesions as shown in Fig. 3. Patients were also examined and evaluated clinically and radiographically with periapical x-rays to assess the healing process at three and six months. Healing was evaluated using the modified Penn 3-dimensional criteria, as described by Schloss et al. and illustrated in Fig. 4.

### Statistical analysis

We assessed the normality of numerical data by examining its distribution and conducting normality tests, including the Kolmogorov–Smirnov and Shapiro–Wilk tests. The age data exhibited a normal distribution, while the lesion size data demonstrated a non-normal distribution. The data was summarized using median, range, mean, and standard deviation (SD) values. For normal data, we employed a one-way ANOVA test to compare mean age values across the three groups. For non-normal data, we utilized the Kruskal–Wallis test to compare between the groups. In intra-group changes, we employed the Wilcoxon signed-rank test to examine changes within each group. Qualitative data were summarized using frequencies and percentages. In the comparison of categorical variables, we used either the Chi-square or Fisher’s Exact test. The level of significance was set at P ≤ 0.05. Statistical analysis was performed using IBM program SPSS Statistics for Windows, Version 23.0 (Armonk, NY: IBM Corp.).

## Results

The recruitment of patients started on 01/01/2020, and the final follow-up was achieved on 30/09/2022, after which the trial was concluded. The flowchart of participants during each phase of the trial, namely enrollment, randomization, allocation, follow-up at 12 months, and data analysis, is depicted in Fig. 1.

Out of the 56 patients enrolled in the study, 7 were lost to follow-up, 2 traveled abroad, 1 died in a road accident, and 4 were unreachable. (1 in the MTA group, 2 in the TotalFill group, 2 in the MTA & bone group, and 2 in the TotalFill & bone group). A total of 49 patients were available for the final analysis. The demographic data of the patients is presented in Table 1. There was no statistically significant difference between gender distributions and age values in the four groups.

**Table 1:** Descriptive statistics and results of Chi-square and one-way ANOVA tests for comparisons between demographic data of the four groups.

Demographic data	TotalFill (n = 14)	MTA (n = 14)	TotalFill with bone graft (n = 14)	MTA with bone graft (n = 14)	*P*-value
Gender [n, (%)]					
Male	6 (42.9%)	8 (57.1%)	6 (42.9%)	6 (42.9%)	0.767
Female	8 (57.1%)	6 (42.9%)	8 (57.1%)	8 (57.1%)	
Age [Mean, SD]	30.5 (7.6)	30.6 (7.1)	31.4 (6.6)	31.3 (7.2)	0.984

Results showed high success rates being achieved using MTA and TotalFill in healing periapical lesions in endodontic surgery. The addition of bone graft in small and medium sized lesions did not affect the success rate of endodontic surgeries.

Changes in lesion size and percentage reduction in lesion size in all four groups are presented in Fig. 3 and Table 2, respectively. A comparison between the healing criteria in each group can be observed in Table 2.

**Table 2:** Frequencies (n), percentages (%) and results of Fisher’s Exact test for comparison between healing criteria in the four groups.

Group	Complete healing		Limited healing		Uncertain healing		Unsatisfactory healing		*P*-value	Effect size *(v)*
	n	%	n	%	n	%	n	%		
TotalFill (n = 12)	4	33.3	6	50	1	8.3	1	8.3	0.999	0.095
MTA (n = 13)	5	38.5	5	38.5	2	15.4	1	8.3		
TotalFill with bone graft (n = 12)	6	50	4	33.3	1	8.3	1	8.3		
MTA with bone graft (n = 12)	5	41.7	5	41.7	1	8.3	1	8.3		

The reduction in lesion size was 72.1%, 67.7%, 65.9%, and 69%, respectively. The addition of bone with MTA showed 9% less reduction in lesion size, while it showed a 2% more reduction when added to TotalFill (Table 3). There was no statistically significant difference between lesion sizes when comparing the four groups, either preoperatively or postoperatively (Table 4). No side effects were reported for any of the materials used in this study.

**Table 3:** Descriptive statistics and results of Kruskal–Wallis test for comparison between percentage reductions in lesion size in the four groups.

Group	Reduction in lesion size (%)	
	Median (Range)	Mean (SD)
TotalFill (n = 12)	69.6 (51.1–93.3)	72.1 (12.5)
MTA (n = 13)	76.7 (12.7–93.5)	67.7 (23.6)
TotalFill with bone graft (n = 12)	73.7 (16.1–88.4)	65.9 (22.8)
MTA with bone graft (n = 12)	73.2 (11.1–92.4)	69 (23.3)
*P*-value	0.980	
Effect size (*Eta Squared*)	0.012

**Table 4:** Descriptive statistics and results of Kruskal–Wallis test for comparison between lesion sizes in the four groups.

Group	Pre-operative		Post-operative	
	Median (Range)	Mean (SD)	Median (Range)	Mean (SD)
TotalFill (n = 12)	137.6 (68.1–264.1)	151 (65.1)	32.9 (10.1–107.4)	42.2 (29.6)
MTA (n = 13)	123.5 (65.3–384.2)	154 (91.4)	34.9 (8.1–180.9)	53.1 (54.1)
TotalFill with bone graft (n = 12)	174.8 (94.6–317.6)	190.3 (58.1)	46 (14.2–221.9)	73.3 (69.5)
MTA with bone graft (n = 12)	204 (71.1–328)	192.8 (88)	45.7 (8.1–97.6)	50.8 (30.1)
*P*-value	0.197		0.620	
Effect size (*Eta Squared*)	0.065		0.055	

## Discussion

Endodontic surgery represents a final chance in trying to save the natural tooth when conventional retreatment has failed or is impossible to be done. The surgical procedures aim to eradicate infected tissues through the resection of the apical portion of the tooth followed by root-end cavity preparation & filling^11^. Patients were treated by the same couple of operators (HS and AS) who were well-trained and experienced, eliminating the learning curve or the impact of the operator qualifications on the outcome.

The strengths of the present study are in the randomization of patients, the study of root-end filling material with or without the addition of bone, triple blinding, providing a satisfactory follow-up period of one full year, and using 3D imaging. To ensure blinding, MTA and TotalFill have similar radiodensity, rendering them radiographically indistinguishable. Accordingly, it takes an average of 4 to 6 months for the bone graft to resorb; thus, at the end of the follow-up period, the assessors cannot discern whether a bone graft was used^9^. The randomization procedure ensures that groups had an even distribution of known and unknown confounding factors.

In the current study, we aimed to evaluate the periapical healing following endodontic microsurgery (EMS) using a new bioceramic material, TotalFill in Putty form, as a bioactive retro-filling material in comparison to the current gold standard material, MTA. We also wanted to evaluate the interaction of these biomaterials with a bioactive composite bone graft combining both xenogeneic and autogenous bone particles to see whether it affected the healing process or not.

Studies proved that the orthograde root filling material (gutta percha) is insufficient alone for decent long-term healing of periapical tissues. Bacterial growth in root canals and their transmission to periapical tissues can only be prevented by sealing the apical portion of the root. Thus, performing and sealing a retro-cavity in a three-dimensional manner using designated instruments and a suitable retro-filling material prevents the recurrence of infection in root canals and preserves the periapical tissues in healthy condition^35,36^.

The type of root-end filling material is one of the variables that impact the outcome of periapical surgery^37^. Ideally, root-end filling materials should provide a bacterial tight seal to the root canal system. They should also be biocompatible, bactericidal, or at least bacteriostatic. They should provide adequate dimensional stability while remaining non-resorbable and bioactive to promote cementogenesis and osteogenesis.

MTA has been the material of choice for root-end filling owing to its biocompatibility, excellent sealing ability, hard tissue induction/conduction, and high rate of success^38,39^. MTA remains the gold standard against which new materials must be tested^7^.

The main drawbacks of the original formula of MTA include a problematically long setting time, difficult handling characteristics, potential of tooth discoloration, presence of toxic elements in the composition, and the difficulty of removal after final setting has occurred^40^. Hence, new formulas and bioactive materials are constantly being introduced to surpass the standard MTA formula, that has been used for the last several decades. Plenty of companies have introduced new CSC which can be used as root-end filling due to their ability to release calcium hydroxide in solution as part of their setting reaction^12^. Such companies include: Septodont Saint-Maur-des-fosses, (France) which introduced Biodentine, Innovative Bioceramix Inc, (Vancouver, Canada) which introduced Bioaggregate, and Brasseler USA (Savannah GA) which introduced EndoSequence Root Repair Material (RRM) which comes in medium and putty consistency.

Bioceramic cements are premixed, homogeneous, and consistent materials. Their physical properties, as per manufacturers’ claims, include exceptional dimensional stability, high mechanical bond strength, high alkaline pH, acceptable radiopacity, and hydrophilic setting properties^41^.

In vitro studies comparing these new materials to MTA conclude that bioceramic materials have comparable levels of cytotoxicity to the gold standard MTA, which makes them highly biocompatible^42^. Other studies compared both antibacterial properties and sealing ability of Bioceramics to that of MTA and concluded that they are mostly similar^43,44^. In an animal model study, CSC even surpassed MTA in tissue healing response that is adjacent to the resected root-end surface using histological examination. This superior healing tendency associated with new CSC could even be detected radiographically using CBCT and micro-CT technologies^45^.

As stated earlier, a distinction has to be made between traditional endodontic surgery and EMS in which magnification in conjunction with coaxial illumination is utilized^18^. In a direct comparison between the success rates of traditional periapical surgery and EMS, authors concluded that the success rates were 59% for traditional surgeries and 94% for modernized EMS techniques^11^. This marked increase in the success rate of EMS is not only attributed to magnification and introduction of new biomaterials for root-end filling but also to the biomaterials used for guided bone regeneration.

The larger the periapical defect, the greater the indication for using guided bone regeneration techniques with biomaterials, such as bone grafts and membranes. These techniques help eliminate non-osteogenic tissue regeneration patterns that would differ significantly from the original structures that once filled the defective area^46^. Complete bone formation of the surgical wound post-endodontic surgery is an essential step in healing, giving rise to various strategies being proposed to stimulate osteogenesis. Autogenous bone graft remains the reference biomaterial for bone repair due to its combined qualities such as osteogenesis, osteo-induction, and osteo-conduction. In addition, autogenous bone graft is non-immunogenic as it is taken from the same subject. However, using homologous bone from another subject of the same species or heterologous bone from a different species has disadvantages, such as immune rejection reactions and the possibility of cross infections. This has driven the development of synthetic alloplastic biomaterials that contribute to bone repair. Synthetic or naturally occurring calcium phosphates, xenogeneic bone, natural or synthetic calcium carbonates, calcium sulfates, and bioactive crystals, together with signaling molecules, improve stem cell survival and direct their differentiation towards specific cell lines. Combining xenogeneic and autogenous bone grafts has been reported in maxillofacial reconstruction, benefitting from the qualities of both graft types. In a study evaluating the effect of combining both xenogeneic and autologous bone grafts at different percentages, it was found that adding Bio-Oss to autogenous bone at different concentrations showed superior dimensional stability at a 25% autologous bone proportion compared to the use of autogenous bone alone. Furthermore, there was no significant difference statistically when the autogenous bone proportion was doubled to 50%^34^.

Two-dimensional imaging with periapical (PA) radiography is insufficiently detailed to detect apical periodontitis and minute changes in periodontal ligament reformation^47,48^. Some studies comparing PA versus CBCT healing after surgery with follow-up periods ranging from 4–12 months postoperatively concluded that CBCT imaging shows lower healing rates than PA radiography during the investigated period^8,49,50^. Consequently, in this study, we chose to use the more accurate CBCT for follow up. As for the evaluation period, we assessed the outcome one year after surgery. However, the five-year prognosis can be predicted from the one-year assessment with an accuracy of 91% to 95%^6,51^. Moreover, a recent study by Song et al.^52^ found no significant difference in the clinical outcome after EMS between the one-year follow-up and follow-up at four years or more. Therefore, the one-year follow-up should be sufficient to predict the long-term outcome of apical surgery in our study.

In the present study, using MTA as a root-end filling material posed a 77% success rate, making it a reliable material for use in endodontic surgeries. This is consistent with Saunders et al.^53^, Von Arx et al.^54^, and Shinbori et al.^55^, who demonstrated 89%, 75.9%, and 92% success rates, respectively. Moreover, Bioceramic Putty showed an 83.3% success rate when used as a root-end filling material, consistent with the findings by Von Arx et al.^56^. Similarly, TotalFill presented a 94.1% success rate when used in EMS.

In the current study, all groups showed no significant differences; however, TotalFill as a root-end filling material showed a higher success rate than MTA. This finding is clinically insignificant, as shown in the findings by Kim et al*.*^57^ and Zhou et al.^58^ These excellent results may be attributed to their biocompatibility, good sealing ability, resistance to blood contamination, and promotion of cell proliferation and mineralization. Conversely, the use of MTA or TotalFill in combination with bone graft showed 83.4% and 83.3% success rates, respectively, consistent with Chen et al.^38^. However, this is statistically insignificant, as the positive results in the MTA group may be due to the unique properties of bone graft, being osteo-inductive/osteo-conductive, allowing bone tissue to form. The bone graft acts as a scaffold on which bone tissue can attach, grow, and regenerate, promoting bone healing acceleration.

For the TotalFill group, adding bone did not affect the success rate. This may be attributed to some the characteristics of CSC in better stimulating bone marker expression compared to the MTA, according to the study by Chen et al.^59^. Bone markers are defined as osteoblast-derived proteins that reflect bone formation. One type of bone marker is osteocalcin, a non-collagenous protein found in bone/dentin. It is secreted by osteoblasts and is thought to play a role in mineralization and calcium ion homeostasis. According to the mentioned literature, CSC produced higher osteocalcin expression than MTA. However, both materials were equal in other markers, such as bone sialoprotein, alkaline phosphatase, and osteopontin. This is contrary to the findings by Goyal et al.^16^, Tanomaru- Filho et al.^60^, and Tsesis et al.^19^, who demonstrated complete healing of large periapical lesions without the need for bone grafts. Taschieri et al.^21^ also concluded that the use of GTR in association with bovine bone in the treatment of these lesions of endodontic origin did not provide benefits in terms of regeneration. Furthermore, a review carried out by Lin et al.^20^ concluded that the application of bone grafts does not ensure complete periapical regeneration, as these materials are not capable of attracting stem cells or stimulating their differentiation into osteoblasts and cementoblasts.

The results of this study support the hypothesis that the addition of bone to small-to-medium size lesions will not significantly enhance the success rate of the EMS procedure. These results coincide with the hypothesis that TotalFill can be used as an alternative to the gold standard MTA, showing similar success rates. Although the success rates were high regardless of bone grafting, larger lesions may require their addition to show similar results. Only single-rooted maxillary teeth in healthy patients were tested, which is a limitation in this study. Furthermore, results on large lesions cannot be inferred from our outcomes, which resulted from testing on small-to-medium sized lesions.

Further clinical studies are required to test the effect of MTA and TotalFill with and without the use of bone grafts in large lesions. Although using Bioceramic materials that set in moist conditions in Apicoectomy procedures is adequate, large proportion packages may cause them to set prematurely. Therefore, dentists should use them in small proportion packages, such as 0.25 g or less.

## Conclusion

Within the limitations of the current study, it can be concluded that TotalFill shows high success rates in EMS, matching the gold standard of MTA. Additionally, the addition of bone graft in small-to-medium size lesions did not affect the success rate of EMS. Our results are consistent with many studies indicating no significant difference in success rates between MTA and TotalFill. They also align with studies showing similar results when adding bone graft. However, our results do not coincide with some studies claiming that bone graft addition has superior effect on the success rate. The observation period of 12 months is considered satisfactory for determining clinical and radiographic tissue changes towards healing.

## Data Availability

The datasets used and/or analyzed during the current study are available from the corresponding author upon reasonable request.
